# Correction of a postpneumonectomy syndrome with congenital pectus excavatum using Ravitch’s procedure and silicone breast implants. Report of a case

**DOI:** 10.1016/j.ijscr.2020.05.044

**Published:** 2020-05-29

**Authors:** Weam Essaleh, Franz Stanzel, Stefan Welter

**Affiliations:** a Lung Clinic Hemer, Department of Thoracic Surgery, Theo-Funccius-Str. 1, 58675 Hemer, Germany; b Lung Clinic Hemer, Department of Pneumology, Theo-Funccius-Str. 1, 58675 Hemer, Germany

**Keywords:** Postpneumonectomy syndrome, Breast implants, Pectus excavatum, Case report

## Abstract

**Introduction:**

Mediastinal repositioning and the use of allogenic implants to obliterate the postpneumonectomy space is the main principle of postpneumonectomy syndrome (PPS) correction. We present a rare case with a PPS in combination with a congenital pectus excavatum. As a pectus excavatus deformity reduces retrosternal space, simple repositioning of the heart is impossible unless combined with a sternum elevation.

**Presentation of case:**

Two years after left sided pneumonectomy a 30 year old female was admitted with progressive exertional dyspnea and stridor and not able to do her basic activities. Chest CT-scan and bronchoscopy revealed severe right main bronchus stenosis, compression of hilar vessels and the presence of a pectus excavatum deformity. A single stage operative correction was performed with sternum repositioning by a Ravitch’s procedure, pericardial fixation to the right sternal edge and obliteration of the left thoracic cavity with two silicone breast implants. All complaints disappeared within 48 h.

**Discussion:**

To the best of our knowledge, this is the first report about successful treatment of PPS aggravated by a preexisting pectus excavatum in an adult patient. The durability and migration of the silicone implants and the volume reduction of the pericardial sac during fixation to the sternum continues to remain a concern.

**Conclusion:**

Single stage correction of PPS and pectus deformity is feasible and seems to be the appropriate treatment for both pathologies.

## Highlights

• This paper demonstrates the repair of a severe left sided postpneumonectomy syndrome (PPS), aggravated by a pectus excavatum deformity.
• The formation of this kind of pathology is very uncommon and therefore this case might be interesting for the readers.
• We present the successful simultaneous repair of both pathologies.
• The highlight of this case is the complete documentation of preoperative pathology, intraoperative findings and the postoperative outcome.

## 1 Introduction

Post Pneumonectomy Syndrome (PPS) is a rare complication after pneumonectomy, it occurs more often in children than adults characterized by extensive mediastinal shift to the empty hemithorax resulting in symptomatic central air way compression and obstruction [[1], [2], [3]]. Commonly patients present with progressive exertional dyspnea, stridor, occasionally dysphagia and recurrent respiratory infections. Usually symptoms arise ranging from weeks to years after pneumonectomy. The diagnosis is based on CT-scans, pulmonary function test (PFT), and bronchoscopy. As this condition is very rare there are only a few case reports and small case series available, treatment strategies are still a matter of debate [2,4]. To our knowledge this is the first report of a left sided PPS case in an adult with successful simultaneous correction of pectus deformity and heart displacement. This work has been reported in line with the SCARE criteria [5].

## 2 Patient information

We report the case of a 30 year old female, with a history of congenital esophageal atresia and tracheoesophageal fistula which was surgically corrected in childhood followed by repeated laparotomy to correct pyloric stenosis. The patient also had congenital pectus-excavatum and suffered from epilepsy. At the age of 19 years she developed tracheal and left main bronchus stenosis at the site of the former tracheoesophageal fistula. Due to recurrent pneumonia and exertional dyspnea, she was referred to our specialized tertiary pulmonary hospital and underwent a series of interventional bronchoscopies and stent-implantations, beginning with a Dumon-stent (4/2010), followed by a Y-Stent (10/2016) and later with resorbable stents requiring occasional granulation tissue ablation. This was in a period from 2010 to 2016 (Fig. 1). Informed consent was given from her to publish this report.

**Fig. 1:**
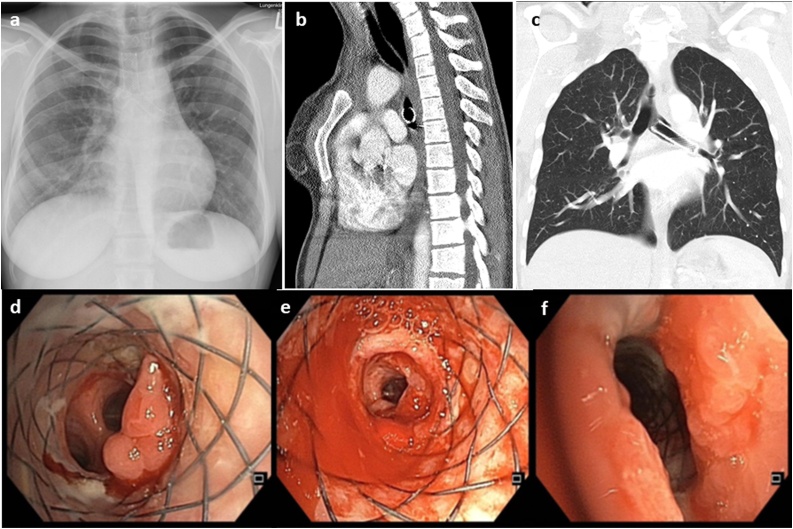
Stent and Pectus excavatum. a) Chest X-ray with a y-stent and bilateral streaky infiltrates and a right sided synostosis of the 5th and 6th rib 2012. b) Sagittal CT-plane demonstrating the pectus deformity of the sternum 2015. c) Coronary CT-plane of the lung with a covered metal stent 2017. d–f) Bronchoscopic views of the last stent with granulation tissue formation at the proximal and distal stent ends and chronic inflammatory changes of the bronchial mucosa.

Finally she underwent elective left pneumonectomy in 7/2017 for destroyed bronchial system and lung. Afterwards the patient recovered well and went through a regular post-operative follow up. No purulent airway infection recurred.

## 3 Clinical findings

After two years she developed PPS with severe left sided mediastinal shift which was probably exaggerated by the congenital pectus-excavatum. She was symptomatic with exertional dyspnea, stridor and finally the inability to perform normal daily activities.

## 4 Diagnostic assessment

The diagnostic work-up included basic blood tests, ECG, pulmonary function tests, flexible bronchoscopy and a CT scan of the chest. Bronchoscopy showed distal tracheal and right main stem bronchus stenosis. The CT-scan revealed filiform stenosis of the right main bronchus and the right veins as well (Fig. 2). So we discussed operative correction of PPS with her.

**Fig. 2:**
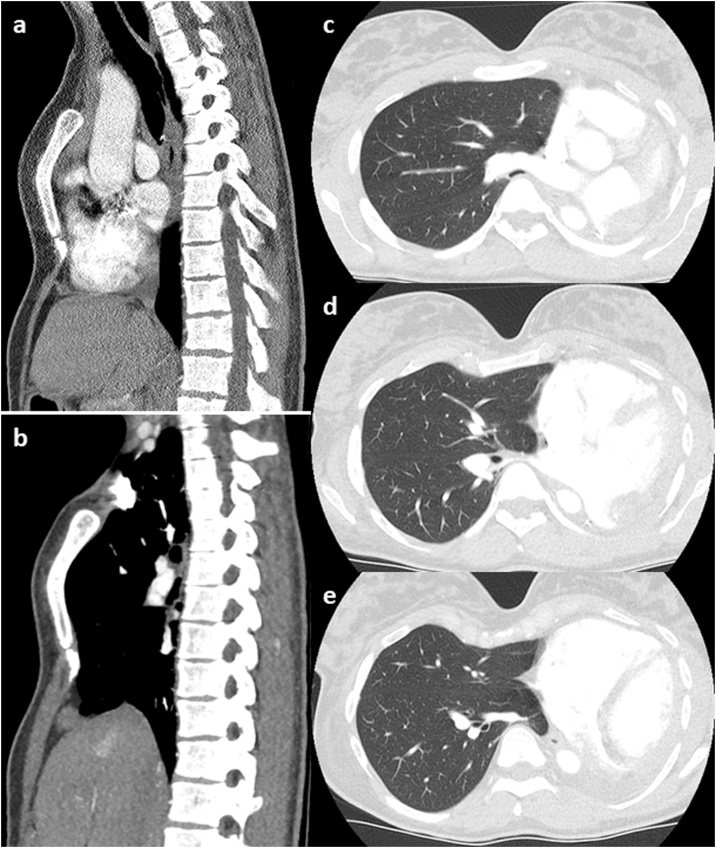
CT status of postpneumonectomy syndrome 6/2019. a) Sagittal CT-scan 2017 demonstrating pectus excavatum prior to pneumonectomy. b) Sagital CT-scan 6/2019 with complete rotation of the heart into the left thoracic cavity. c–e) CT-scans showing the reasons for postpneumonecotmy complains: compression of the right main bronchus posterior to the right pulmonary artery (c), stretching and compression of the right lower pulmonary vein inferior to the right main bronchus (d), minimum distance between posterior border of the sternum und anterior border of the column, not allowing simple repositioning of the heart retrosternally (e).

## 5 Therapeutic intervention

The operation was performed 6/2019 by an experienced thoracic surgeon in supine position, using single lumen tube intubation. Following preoperative planning, correction of both pathologies was performed in four steps (Fig. 3):

1. Repeated left sided thoracotomy and adhesiolysis of the diaphragm, mediastinum and the heart
2. Submammarian incision, intra perichondral resection of the cartilages of the third to eighth rib bilateral, double osteotomy of the sternum, sternum elevation and stabilization with a pectus bar
3. Reposition of the heart using non-resorbable polypropylene sutures through the pericardium, that were fixed at the right sided perichondral pipes
4. Obliteration of the left thoracic cavity with two silicone breast implants of 250 g and 460 g.

**Fig. 3:**
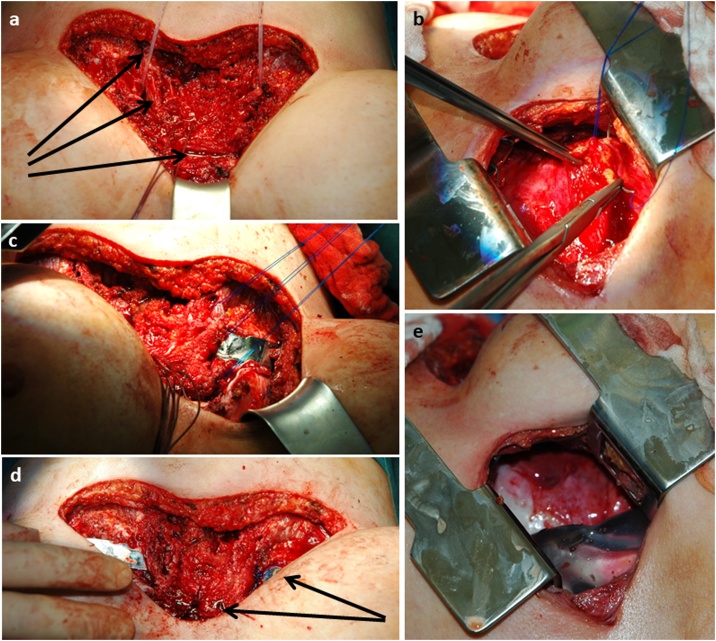
Operative correction of pectus and mediastinal repositioning. The picture demonstrates several steps of the operation: a) horizontal incision in both submammarian folds. The arrows top down show a rubber band to elevate the sternum, the perichondral tubes after removal of the rib cartilages and the horizontal V-osteotomy of the sternum. b) antero-lateral repeat thoracotomy and preparation of 3 pericardial U-stiches with non-resorbable polypropylene size 1 sutures for later heart repostitioning. c) sternal elevation with pectus bar was performed and the 3 polypropylene sutures are pulled from the left side through the right intercostal spaces inside out and later fixed to the perichondral tubes and connective tissue. d) arrows top down: visible small piece of the right pectus bar with the knots of polypropylene sutures after repositioning of the heart and pericardium and the knots, placed through the anterior sternal plate at the site of the osteotomy. e) One of two silicone breast implants filled into the left empty thoracic cavity after extensive antiseptic rinsing.

She was extubated in the operation room and postoperative pain was controlled with peridural anesthesia. Perioperative intravenous cephalosporine antibiotics were given for 3 days and low dose subcutaneous low molecular weight heparine for 10 days.

## 6 Follow-up and outcome

After intensive post-operative physiotherapy the patient had an unremarkable course of recovery and was discharged at postoperative day 12 in good general condition. Endoscopy control revealed patent tracheal and main bronchial lumen with no obstruction at all. No stridor was present and she was very satisfied with the result. (Fig. 4).

**Fig. 4:**
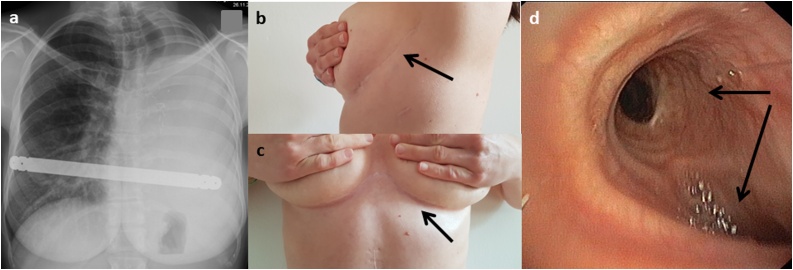
Postoperative results. a) Chest X-ray showing the mediastinum slightly rotated to the left, the pectus bar in correct position. b) Scar of the anterolateral thoracotomy. c) Scar of the submammarian incision to correct the pectus deformity. d) Bronchoscopic views showing open tracheal lumen and no compression of the right main bronchus. Black arrows showing the right main bronchus and the left main bronchus stump.

After 4, 9 and 11 months she was seen in the outpatient clinic for regular follow up. The patient had complete remission of exertional dyspnea and stridor, reduction of pain at the area of the Pectus bar excellent scar healing, a perfect correction of her pectus deformity and she reported being able to perform her normal daily activities with no difficulties. We plan to remove the bar after 24 months. concerning the as well as her gain in quality of life.

## 7 Discussion

Post pneumonectomy syndrome (PPS) is a rare late complication after pneumonectomy independent of the indication. It was described after lung destruction from TBC as well as after tumor resection [1,2,6]. Hyperinflation of the remaining lung, resorption of the serothorax and the elasticity of the mediastinum allowing the rotation of the heart into the empty space, are all factors that contribute to PPS [4,6]. After left pneumonectomy, the mediastinum shifts to the left hemithorax, which results in a clockwise (CT imaging) rotation of the heart and the right main stem bronchus becomes stretched over the anterior vertebral bodies [2,6]. Longer persistence of this state may result in bronchomalacia which is difficult to correct later. Furthermore the right pulmonary artery and veins may become jammed between trachea and the ascending aorta. This can be aggravated by a preexisting pectus excavatum [7]. Left sided PPS has been described only in patients with right aortic arch [2] so pectus excavatum may be the most important factor aggravating symptoms of PPS after left sided pneumonectomy.

Patients with PPS commonly complain about a slow deterioration of their general condition and increasing shortness of breath on exertion as well as a stridor, as was the case in our patient. Other symptoms can vary from dysphagia, syncope and even signs of cardiogenic shock according to the severity of the mediastinal shift. This can be a life threatening situation requiring prompt surgical correction [2,6,7]. The onset of symptoms is within 2 years after pneumonectomy in many patients so it was in our patient as well [6].

In our above mentioned case the PPS was complicated with a congenital pectus excavatum which made the case more challenging. The presence of pectus excavatum in a PPS is very rare and there are very limited publications about surgical correction [7,8]. The reported case of PPS with pectus excavatum was a child that had pleural prothesis in the age of two years and developed pectus deformity and prothesis dislocation 4 years later. The correction was performed through a sternotomy to mobilize the mediastinal contents and fix the pericardium. A further horizontal incision allowed the removal of pathologic costochondral cartilages. Two stainless steel plates were placed across the anterior chest wall underneath the sternum to protect the result [8]. In an earlier case with PPS and heart failure we performed pectus correction only, which led to a relief of most complains [7]. We used open pectus deformity correction with respect of the age of the patient and the associated rigidity of the chest skeleton. Fixation of the pericardium to the sternum after heart repositioning was former described by Grillo H, et al. [2] with special care to avoid reducing the volume of the pericardial sac. Whereas others did not perform any fixation at all in their patients [6]. As there is increased risk for PPS recurrence anyway, the pleural space needs to be filled with allogenic material [1,9].

In our current case the correction was accomplished through a left repeat thoracotomy to reposition the mediastinal structures and perform cardiopexy with non-resorbable sutures. Afterwards a modified Ravitch correction using a submammarian incision was efficiently used to correct the deformity. As reported from others, the thoracic cavity was filled with silicone breast implants [2,3,6,9]. We think our approach had satisfying results surgically as well as aesthetically (Fig. 4). Relieve of bronchial stricture was reported in 8/8 cases after prosthesis insertion [2] and others conclude that mediastinal repositioning with an intrathoracic prosthesis is the treatment of choice for PPS [6].

The durability and migration of the silicone implants continues to remain a concern. Ruptures of saline filled implants as well as rupture of silicone implants were reported [3,4]. Covered and uncovered bronchial stumps were identified as local risk factors. As a solution, the use of a custom expander with wall thickness three times that of a standard expander was suggested [3]. Another concept is to cover the pericardium with intercostal muscle and posterior perichondrium of 4 neighbored ribs to protect it from foreign material contact [2]. But the authors already discussed that muscle flaps may be unnecessary when safe protheses are used.

Satisfactory outcome reports increased over time and reached 90–100% (9/10 patients) in recent reports [4,10], whereas a former series reported 4/11 deaths after PPS correction [2].

## 8 Conclusion

PPS is rare and unpredictable. It can occur after right or left pneumonectomy. Symptoms are manifested due to the shift of mediastinum, leading to compression and stretching of mediastinal structures, the tracheobronchial tree and the esophagus and induce shortness of breath, stridor and heartburn. Pectus excavatum can aggravate the symptoms. Diagnosis must be made by exclusion. A surgical correction is the most effective and efficient treatment on the long term and include mediastinal repositioning, possible pericardiopexy and prosthesis insertion into the empty pleural space. If additional pectus excavatum is present, its correction is crucial to increase retrosternal space and allow pericardial repositioning.

## Declaration of Competing Interest

There are no conflicts of interest.

## Sources of funding

This report has not received any funding.

## Ethical approval

The institutional review board accepted this Case report for publication.

## Consent

The patient did not only give written consent, she also reviewed the manuscript and pictures and accepted to publish all given details.

## Author contribution

Conception and design of the study: WE, SW.

Data acquisition: WE, SW.

Drafting and revising of the article: SW, FS, WE.

Final approval of the version to be submitted: SW, FS, WE.

## Registration of research studies

NA.

## Guarantor

Stefan Welter, MD.

## Provenance and peer review

Not commissioned, externally peer-reviewed.
